# Network Pharmacology-Based Prediction and Verification of the Potential Targets of Pinoresinol Diglucoside for OA Treatment

**DOI:** 10.1155/2022/9733742

**Published:** 2022-04-16

**Authors:** Hongkan Lou, Yang Zhang, Jianli Fang, Yong Jin

**Affiliations:** ^1^Ningbo Hospital of Traditional Chinese Medicine Affiliated to Zhejiang Chinese Medical University, Ningbo 315012, China; ^2^Zhejiang Academy of Traditional Chinese Medicine, Hangzhou 310000, China

## Abstract

**Objective:**

This study aimed to explore the effects and related mechanisms of pinoresinol diglucoside (PDG) on osteoarthritis (OA) via a combination of pharmacology and animal experiments.

**Methods:**

Traditional Chinese Medicine Database and Analysis Platform (TCMSP) Drugbank, Online Mendelian Inheritance in Man, and GeneCards databases were used to predict the putative targets of PGD against OA. A protein protein interaction (PPI) network was constructed in STING database to analyze the interaction network of these targets. Enrichment analysis was performed with DAVID database. The OA model was built by anterior cruciate ligament transection and then injected with PDG for 5 weeks. Hematoxylin and eosin (HE) staining and safranin-fixed green staining were used to evaluate the pathological change. ELISA was applied to measure the serum levels of interleukin-1*β* (IL-1*β*), interleukin-6 (IL-6), and tumor necrosis factor-*α* (TNF-*α*). Immunohistochemistry was employed to detect the protein levels of kinase B (AKT), BAX, Bcl2, matrix metalloproteinase-1 (MMP-1), tissue inhibitor of metalloproteinases 1 (TIMP-1), and phosphatidylinositol 3 kinase (PI3K) in knee cartilage tissues.

**Results:**

Seventy-one key targets were identified, including AKT1, epidermal growth factor receptor, SRC, estrogen receptor 1 (ESR1), and MMP9. Enrichment analysis revealed a series of pathway related to cancer, PI3K-Akt signaling pathway, and proteoglycans in cancer. Animal experiments showed that PDG alleviated the abnormal histomorphological changes of OA; suppressed TIPM, serum IL-1*β*, IL-6, and TNF-*α* levels, and PI3K and AKT activation; and increased MMP-1 expression and Bcl2/Bax ratio.

**Conclusion:**

PDG has a cartilage-protecting effect associated with PI3K/AKT signaling pathway in rabbit with OA and therefore might serve as a potential agent for the treatment of this disease.

## 1. Introduction

Osteoarthritis (OA) is the most common chronic joint disease characterized with the degeneration of articular cartilage and the osteogenesis of articular margin and subchondral bone [1]. Chondrocyte reduction in articular tissues, metabolic disorder of extracellular matrix (ECM), inflammatory reaction of the synovial membrane of articular tissues, and remodeling of subchondral bone eventually lead to joint deformation, joint dysfunction, and even long-term disability in patients with OA [2, 3]. Approximately, 302 million people worldwide are affected by OA [4], and the prevalence increases with age, especially for individuals over the age of 65 years [5]. With population aging, OA consumes a substantial amount of healthcare resources and costs [6]. Its current treatment mainly includes nonsteroidal anti-inflammatory drugs and sodium hyaluronate that only have limited therapeutic effects and produce side effects [7, 8]. Therefore, the development of effective and safe therapies is increasingly important.

*Eucommia ulmoides* Oliver, a traditional medicine used in China, is usually recommended for reinforcing muscles and lungs and lowering blood pressure [9]. This plant could inhibit OA development by improving cartilage metabolism, regulating the extracellular matrix degradation in the articular cartilage, and inhibiting apoptosis in chondrocytes [10]. A clinical trial also provided evidence of the efficacy and safety of its extract as OA treatment [11]. Pinoresinol diglucoside (PDG), a lignanoid, is the main active compound of *E. ulmoides* Oliver [12]; however, its therapeutic effects on OA have not been reported. This research utilized network pharmacology to investigate the potential role and related mechanism of PDG in OA treatment. A rabbit OA model was also established to further verify its pharmacology effects.

## 2. Materials and Methods

### 2.1. Network Pharmacology Analysis

#### 2.1.1. Reverse Target Seeking Calculation

The chemical structural formula of PDG was downloaded from Traditional Chinese Medicine Database and Analysis Platform (TCMSP, https://tcmsp-e.com/) and saved as a file in SDF format, which was then imported to Discovery Studio 4.5 (DS) for reverse targeting. First, the pharmacophore modules of DS were opened. Second, the database of pharmaDB pharmacophores was employed as a ligand profiler for reverse targeting, and all parameters were set as default.

#### 2.1.2. OA Target Collection

Online Mendelian Inheritance in Man (https://omim.org/) and GeneCards (https://www.genecards.org/) databases and Drugbank (https://www.drugbank.com/) were searched using “Osteoarthritis” keyword to identify the corresponding targets of OA.

#### 2.1.3. Venn Diagram Mapping

After OA and PDG target collection, all targets were inputted to imageGP (http://www.ehbio.com/) to obtain the common targets via Venn diagram.

#### 2.1.4. Protein-Protein Interaction (PPI) Network Construction

The common targets of OA and PDG were inputted to String 11.5 (https://string-db.org/), with the organism “Homo sapiens”, to systematically understand and obtain relevant information on protein interaction. A PPI network was then constructed by Cytoscape 3.5.1 software for visual analysis.

### 2.2. Gene Ontology (GO) Functional Enrichment and Kyoto Encyclopedia of Genes and Genomes (KEGG) Pathway Analysis

Targets were inputted in DAVID for GO functional enrichment, including biological processes (BPs), cell component (CC), and molecular function (MF). The top 10 items were shown as histogram using GraphPad Prism 8 software. KEGG pathway was used to analyze the biological pathways of related proteins which is shown as a bubble chart in imageGP (http://www.ehbio.com/).

### 2.3. Animal Experiment

#### 2.3.1. Animal Treatment

Forty New Zealand Rabbits (weight of approximately 2 kg), half male and half female, were bought from Animal Center of Zhejiang University and used to build an OA model after adaptive feeding for 7 days. First, the animals were anesthetized with 4% pentobarbital sodium (1 mL/kg) and then underwent anterior cruciate ligament transection [13, 14]. The anterior drawer test verified that the anterior cruciate ligament was completely severed. Each rabbit was intramuscularly injected with 400000 U penicillin 3 days after operation to prevent infection. The 32 surviving rabbits were randomly and equally divided into model group, sodium hyaluronate injection (Shandong Bauslun Freida Pharmaceutical Co. LTD, Shangdong, China) group (SH, injection volume: 0.3 mL), and PDG group (2 mg/mL, high, medium, low dose, injection volume: 0.4, 0,1 mL). PDG (Solarbio, Beijing, China) was dissolved in normal saline at a concentration of 2 mg/mL. The control group was treated with sham operation. After 4 weeks of modeling, the SH and PDG groups received intra-articular injection with SH and PDG, respectively, once a week for 5 weeks. One week after the final intra-articular injection, the knee cartilage, and serum of each rabbit were collected and stored at −80°C.

#### 2.3.2. Hematoxylin and Eosin (HE) Staining and Safranin-Fixed Green Staining

Knee cartilage tissues were fixed in 10% formalin and embedded in paraffin. HE staining and safranin-fixed green staining were used to evaluate the histopathological changes of knee cartilage tissues. Sections were visualized using a Nikon Eclipse E600 microscope (Kawasaki, Kanagawa, Japan) at 100 × magnification.

#### 2.3.3. ELISA

IL-1*β*, IL-6, and TNF-*α* serum expression levels were detected using ELISA kits (BOSTER, Hubei, China) in accordance with the manufacturer's protocol. Absorbance was measured at 450 nm using a microplate reader (Bio-Rad 680, Bio-Rad, Hercules, CA, USA).

#### 2.3.4. Immunohistochemistry

AKT, BAX, Bcl_2_, MMP-1, TIMP, and PI3K levels were detected by immunohistochemistry. After being dewaxed in xylene, hydrated through an alcohol gradient, antigen retrieval, blocked in BSA, the paraffin sections were incubated with primary bodies (1 : 200, Proteintech, Hubei, China) for overnight at 4°C and then treated with a polyperoxidase-anti-mouse/rabbit IgG secondary antibody (BOSTER, Hubei, China) for 2 hours at room temperature. The DAB kit was used to develop staining. The sections were imaged using an electron microscope (Nikon, Japan) and analyzed using Image-Pro Plus software for semiquantitative analyses.

### 2.4. Statistical Analysis

All values were expressed as mean ± SD. All statistical analyses were carried out with SPSS 20.0 (IBM Corp., Armonk, NY, USA). Two-tailed Student's *t*-test or one-way ANOVA was used for normally distributed data comparison. Differences were considered statistically at a *P* value < 0.05.

## 3. Results

### 3.1. Common Targets of the Compound and Disease

**Figure 1:**
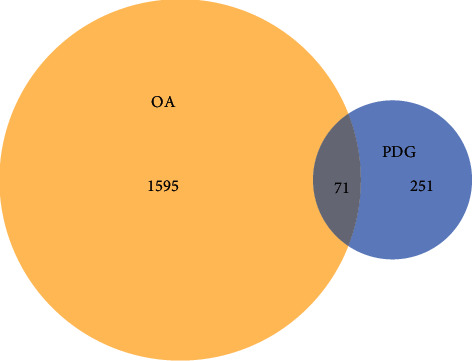
Venn diagram.

A Venn diagram was drawn to find the common targets of PDG and OA. A total of 322 and 1716 targets were found for PDG and OA, respectively, and the overlapping targets were 71 (Figure 1).

### 3.2. Analysis of the Common Targets in the PPI Network

**Figure 2:**
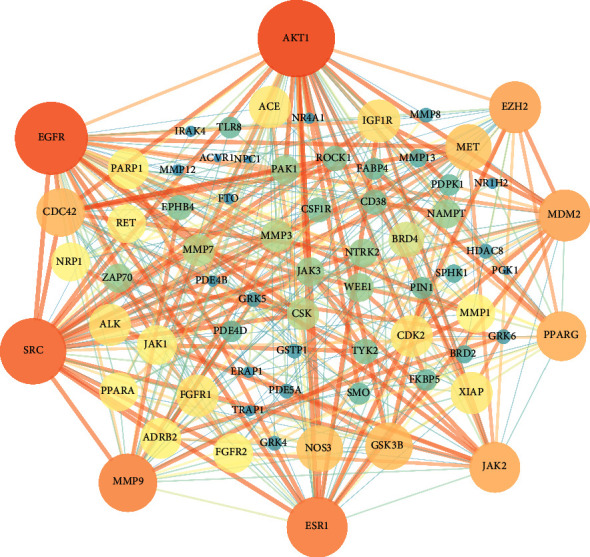
Protein-protein interaction network.

A PPI network was constructed to analyze the underling mechanisms of PDG as OA treatment. As shown in Figure 2, the network has 71 nodes, 350 edges, and an average node degree of 9.86. After topology analysis by Cytoscape software, targets including AKT1 (degree value = 45), EGFR (degree value = 41), SRC (degree value = 35), ESR1 (degree value = 30), MMP9 (degree value = 28), EZH2 (degree value = 22), MDM2 (Degree value = 22), JAK2 (degree value = 21), and PPARG (degree value = 20) had higher degree than others and are mainly involved in cell survival and apoptosis.

### 3.3. Functional Enrichment Analysis

**Figure 3:**
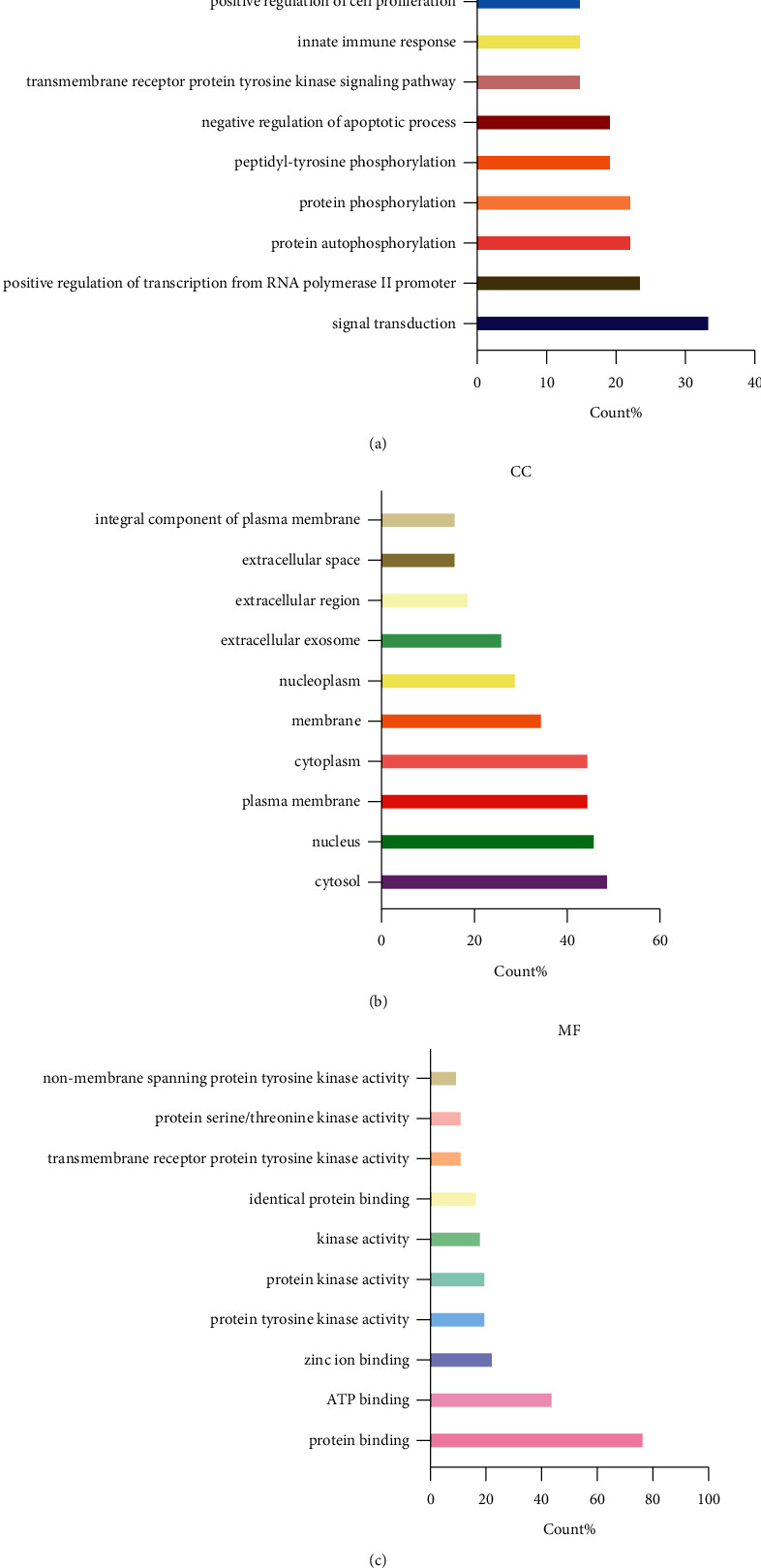
Gene function GO enrichment. (a) Biological process (BP, Top 10); (b) Cellular component (CC, Top 10); (c) Molecular function (MF, Top 10).

The common targets of PDG and OA were inputted to DAVID database to perform GO functional enrichment analysis including BP, CC, and MF. The top 10 items were selected for visualization (Figures 3(a)–3(c)). The results showed 220 terms of BP, such as signal transduction and negative regulation of apoptotic process; 31 terms of CC, including cytosol and nucleus; and 58 terms of MF, such as protein binding and ATP binding.

**Figure 4:**
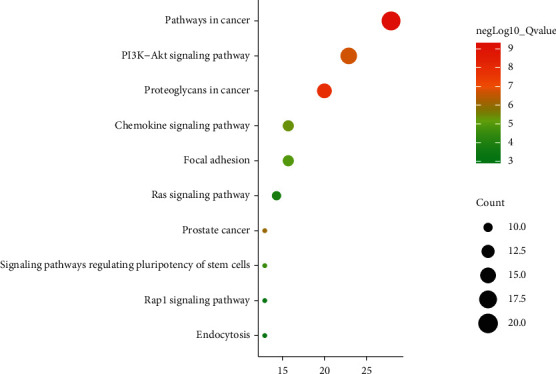
Gene KEGG pathway enrichment. PDG alleviated the abnormal histomorphological changes of OA.

KEGG pathway analysis identified 49 pathways that were significantly enriched (*P* value < 0.05). The top 10 items are shown in Figure 4. The related pathways mainly include cancer, PI3K-Akt signaling pathway, and proteoglycans in cancer. Therefore, the mechanism of PDG for OA treatment might be related to cell apoptosis.

**Figure 5:**
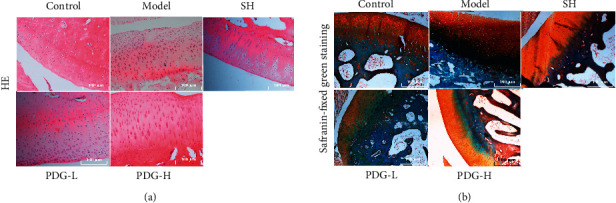
PDG reversed the histomorphological changes of OA. The slides of knee cartilage tissue (*n* = 8) for each group were stained with (a) hematoxylin-eosin (HE) and (b) safranin-fixed green staining.

The defect and thinning of articular cartilage layer, loss of whole cartilage layer in some areas, and severe damage of tangent layer were observed in the model group. After PDG treatment, HE staining images showed the smooth surface of articular cartilage and the chondrocytes orderly arranged with a definite boundary and clear tidal line. Therefore, this compound reversed the abnormal phenomena in OA (Figure 5(a)). Safranine O is a basic dye that colors the cartilage matrix red, and fast green is an acidic dye that colors the surface of cartilage and subchondral bone green. As shown in Figure 5(b), the structure of the four layers of cartilage was clear. In the control group, Safranine O stained the matrix pink, and fast green stained the superficial layer and subchondral bone green. However, in the model group, the superficial surface of articular cartilage was rough, and large cracks were found on the cartilage surface. These structures were improved after PDG treatment.

**Figure 6:**
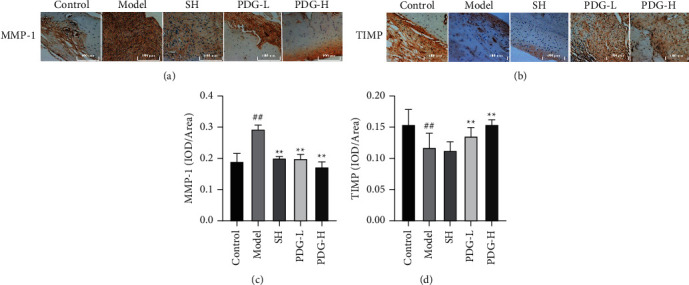
Effect of PDG on MMP-1 and TIMP expression. (a) MMP-1 and (b) TIMP levels were measured by immunohistochemistry. Image-Pro Plus software was used for the semiquantitative analyses of (c) MMP-1 and (d) TIMP. The values are expressed as mean ± standard deviation (SD). ^##^*P* < 0.01 versus control group and ^*∗∗*^*P* < 0.01 versus model group. PDG ameliorated OA via the PI3K/AKT pathway.

MMP/TIMP balance is disrupted by the inflammatory factor in cartilage tissues and ultimately leads to OA. Compared with that in the control group, MMP-1 expression increased (0.1903 ± 0.0258 vs. 0.2938 ± 0.0129) and that of TIMP decreased in the model group (0.1545 ± 0.0239 vs. 0.1176 ± 0.0226). PDG reversed the changes in MMP-1 (PDG-L: 0.199 ± 0.0139; PDG-H: 0.1726 ± 0.0163) and TIMP (PDG-L: 0.1358 ± 0.0136; PDG-H: 0.1543 ± 0.0076) levels (*P* < 0.01, Figures 6(a)–6(d)).

**Figure 7:**
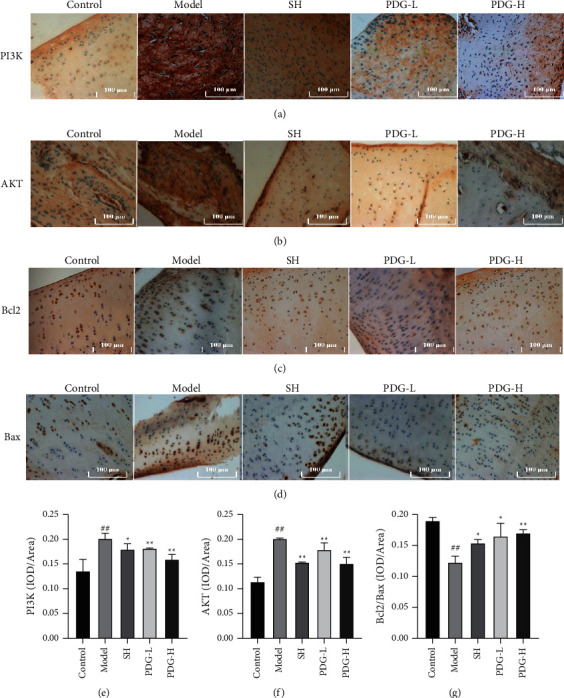
Effect of PDG on the expression of PI3K, AKT, Bcl2, and Bax. (a) PI3K, (b) AKT, (c) Bcl2, and (d) Bax levels were measured by immunohistochemistry. Image-Pro Plus software was used for the semiquantitative analyses of (e) PI3K, (f) AKT, and (g) Bcl2/Bax ratio. The values are expressed as mean ± standard deviation (SD). ^##^*P* < 0.01 versus control group, ^*∗*^*P* < 0.05^*∗∗*^*P* < 0.01 versus model group. PDG inhibited inflammatory response in OA model.

PPI and KEGG results revealed that the PI3K/AKT pathway could be the underlying mechanism of PDG in OA treatment. Hence, PI3K and AKT expression levels in knee cartilage tissues were measured by immunohistochemistry. The data showed that PDG decreased their levels, which were increased in the OA group (PDG-H vs. model group: 0.1595 ± 0.0102 vs. 0.2012 ± 0.0112 (PI3K); 0.1511 ± 0.0123 vs. 0.2124 ± 0.0012 (AKT), *P* < 0.05, 0.01, Figures 7(a), 7(b), 7(e), and 7(f)). PI3K/AKT pathway plays an important role in cell apoptosis by regulating related proteins such as Bcl2 and Bax. As shown in Figures 7(c), 7(d), Bax expression was reduced and that of Bcl2 was enhanced after the OA model was treated with PDG. Compared with that in the control group, Bcl2/Bax ratio was reduced in the model group and reversed in the PDG group (Control vs. model vs. PDG-H group: 0.1901 ± 0.0052 vs. 0.1227 ± 0.0099 vs. 0.1701 ± 0.0052, *P* < 0.05, 0.01, Figure 7(g). These results showed that PDG ameliorated OA possibly via inhibiting PI3K/AKT pathway activation.

**Figure 8:**
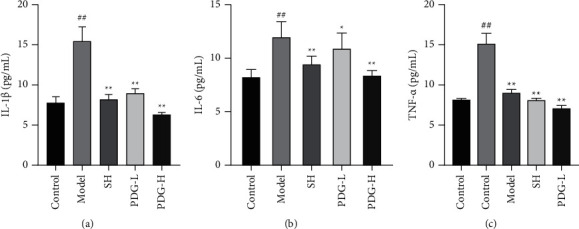
Effect of PDG on IL-1*β*, IL-6, and TNF-*α*. (a) IL-1*β*, (b) IL-6, and (c) TNF-*α* levels were measured using ELISA kits. The values are expressed as mean ± standard deviation (SD). ^##^*P* < 0.01 versus control group, ^*∗*^*P* < 0.05^*∗∗*^*P* < 0.01 versus model group.

OA is an inflammatory joint disease, and the production of inflammatory cytokines is associated with the PI3K/AKT signaling pathway. Therefore, the serum levels of IL-1*β*, IL-6, and TNF-*α* were detected using ELISA kits. PDG treatment inhibited the productions of IL-1*β*, IL-6, and TNF-*α*, which were significantly higher in the model group (PDG vs. Model group: 6.33 ± 0.27 vs. 15.5 ± 1.76; 8.37 ± 0.50 vs. 12.0 ± 1.43, 6.45 ± 0.33 vs. 9.12 ± 0.35) than in the other groups (*P* < 0.05, 0.01, Figures 8(a)–8(c)).

## 4. Discussion

Network pharmacology-based approach and in vivo experimental validation were adopted to reveal the pharmacology effect and molecular mechanism of PDG for OA treatment. PPI analysis showed 71 overlapping targets of OA and PDG. KEGG pathway enrichment also indicated that PDG ameliorate OA via pathways in cancer, PI3K-Akt signaling pathway, and proteoglycans in cancer. In vivo experiment showed that PDG suppressed inflammatory response and cell apoptosis to alleviate OA aggravation by inhibiting the PI3K/AKT signaling pathway.

OA is a degenerative joint disease characterized by cartilage degeneration, and most currently available drugs only temporarily target pain relief and fail to prevent disease progression [15, 16]. Therefore, safe and effective agents to extenuate OA deterioration must be developed. PDG is one of the major lignans isolated from *E. ulmoides* Oliver, which has extensive pharmacological activities, such as antihypertensive and antioxidative [17–19], and is the most commonly prescribed single herb for OA treatment [20]. This work further explored the effect of PDG on OA development. The results of HE staining and safranin-fixed green staining confirmed that PDG reversed the abnormal phenomena in the model group, namely, the loss of whole cartilage layer in some areas, severe damage of tangent layer, and rough superficial surface of articular cartilage. Hu used histomorphometry and showed that loganin delays OA progression [21]. The balance of TIMP and MMP is critical in normal cartilage [22]. Decreased TIMP levels can increase MMP activity, which in turn accelerates ECM degradation and ultimately promotes OA [23]. Animal experiments further showed decreased MMP-1 level and improved TIMP-1 expression in cartilage after PDG treatment.

PPI network revealed that AKT1, EGFR, SRC, ESR1, MMP9, EZH2, MDM2, JAK2, PPARG, and especially AKT played a core role in the mechanism of PDG for OA treatment. KEGG results also showed that the PI3K/AKT pathway could also be implicated. AKT regulates biological functions, such as cell survival and proliferation, and is the vital messenger in PI3K signaling [24, 25]. In OA development, PI3K/AKT pathway activation decreased the apoptosis of chondrocytes with decreased Bcl2 and increased Bax [26]. Guowang Zhang et al. established OA models of mice with articular cartilage loss and found that curcumin reduced cell apoptosis to alleviate OA, and this function is associated with the inhibition of PI3K/AKT pathway [27]. In the present study, PI3K and AKT expression levels were increased in the OA model, and PDG reversed this tendency. In additional, PDG treatment reduced the cell apoptosis of OA model by upregulating Bcl2/Bax expression.

Inflammation response always occurs with OA pathogenesis and OA-related symptoms and is mediated by PI3K/AKT, inflammation cytokines, such as IL-1*β*, accelerate cartilage degradation [28–30]. Bao et al. revealed that patients with OA showed significantly increased expression of AKT, PI3K, IL-6, and TNF-*α* compared with healthy individuals [31]. Therefore, PDG possibly suppresses the inflammation response in OA via the PI3K/AKT pathway.

## 5. Conclusion

**Figure 9:**
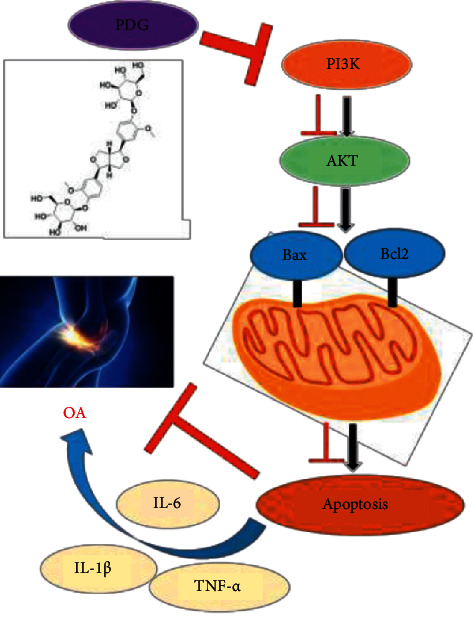
PDG treated with OA via PI3K/AKT signaling pathway.

PDG has a cartilage-protecting effect in rabbits with OA potentially by regulating the ECM degradation of the articular cartilage and suppressing inflammation response and apoptosis in chondrocytes. This function is possibly associated with the PI3K/AKT signaling pathway (Figure 9).

## Data Availability

The data used to support the findings of this study are included in the article.

## Abbreviations

OA: Osteoarthritis
TCMSP: Traditional Chinese medicine database and analysis platform
PPI: Protein-protein interaction network
IL-1*β*: Interleukin-1*β*
IL-6: Interleukin-6
TNF-*α*: Tumor necrosis factor-*α*
MMP-1: Matrix metalloproteinase-1
TIMP-1: Tissue inhibitor of metalloproteinases 1
PI3K: Phosphatidylinositol 3 kinase.
